# Epidermal Growth Factor Receptor Tyrosine Kinase Inhibition Up-regulates Interleukin-6 in Cancer Cells and Induces Subsequent Development of Interstitial Pneumonia

**DOI:** 10.18632/oncotarget.939

**Published:** 2013-03-31

**Authors:** Yukari Ishiguro, Hitoshi Ishiguro, Hiroshi Miyamoto

**Affiliations:** ^1^ Department of Biology and Function in the Head and Neck, Yokohama City University Graduate School of Medicine, Yokohama, Kanagawa, Japan; ^2^ Department of Urology, Yokohama City University Graduate School of Medicine, Yokohama, Kanagawa, Japan; ^3^ Department of Pathology and Laboratory Medicine, University of Rochester Medical Center, Rochester, New York

**Keywords:** fibrosis, EGFR-TKI, IL-6, cytokine, side-effect

## Abstract

Acute interstitial pneumonia is one of serious side effects of epidermal growth factor receptor (EGFR)-tyrosine kinase inhibitor (TKI) treatment, while it often has significant clinical benefit in cancer patients. Therefore, it is necessary to clarify underlying mechanisms for the development of the adverse effects by EGFR-TKI. In the present study, we attempted to determine how EGFR-TKI treatment in cancer cells induced interstitial pneumonia. The growth of tongue cancer HSC-3 and lung cancer A549 cell lines treated with EGFR-TKI was assessed by MTT assay. Cytokines and growth factors in conditioned medium obtained from EGFR-TKI-treated cancer cells were analyzed using cytokine membrane array and ELISA. Interleukin-6 (IL-6) promoter activity was measured by luciferase assay. We found that EGFR-TKI treatment significantly decreased the cell viability yet increased expression levels of IL-6 protein and mRNA, IL-6 secretion, and IL-6 transcriptional activity in these lines. In addition, using the co-culture model and IL-6 treatment was found to increase the expression of collagen and α-actin, which were markers for fibrosis, in lung fibroblast cells. These results suggest that up-regulated IL-6 plays an important role in the development of EGFR-TKI-induced interstitial fibroblastic proliferation. Therefore, blocking of IL-6 signaling could be beneficial to cancer patients undergoing EGFR-TKI treatment for reducing the risk of its unfavorable effects.

## INTRODUCTION

Epidermal growth factor (EGF) is a well-known growth factor that promotes cancer progression. EGF stimulates cancer growth through the EGFR receptor (EGFR) pathway. Most of cancer tissues are shown to overexpress EGFR, and EGF-EGFR axis has been considered a attractive target for cancer treatment. Indeed, EGFR-tyrosine kinase inhibitors (EGFR-TKIs) have been developed and clinically used in patients with malignancy, especially non-small cell lung carcinoma [1,2]. EGFR-TKI effectively blocks the EGFR pathway via suppression of EGFR phosphorylation and can therefore inhibit the growth of cancer cells dramatically. On the other hand, the effect of EGFR-TKI is dependent on mutations of *EGFR* and *KRAS*, *MET* amplification, and the interaction between EGFR and HER-2 and so on [2-6]. More importantly, EGFR-TKI treatment gives rise to severe side effects, including acute interstitial pneumonia [7]. Although some studies have suggested risk factors for side effects [8-12], detailed molecular mechanism for their development remains unknown.

Recently, Kim *et al.* indicated that EGFR-TKI activated STAT3 in non-small cell lung cancer cells [13]. They also showed that STAT3 activation was caused by interleukin-6 (IL-6) in an autocrine manner. IL-6 is one of inflammatory cytokines and is well known as a cancer progression-related cytokine [14,15]. Because STAT3 is one of the targets for anti-cancer drug resistance [16], most of investigations have been only focused on how IL-6 regulates the drug resistance in EGFR-TKI-treated cancer cells.

In the current study, we explored therapeutic effects of EGFR tyrosine kinase inhibition, using two EGFR-TKIs and an αEGFR antibody, in human tongue and lung cancer cell lines. Further, we found that EGFR blocking could increase IL-6 in the cancer cells. Because IL-6 has been suggested to contribute to the development or progression of acute interstitial pneumonia [17-20], we anticipated the possible linkage between IL-6 from cancer cells and EGFR-TKI-induced acute interstitial pneumonia. Our results suggested that IL-6 secreted from EGFR-TKI-treated cancer cells induced lung fibrosis. Accordingly, a combination of IL-6 pathway blocker and EGFR-TKI may show more favorable effects in cancer patients.

## RESULTS

### EGFR-TKI inhibits the growth of cancer cell lines

**Figure 1:**
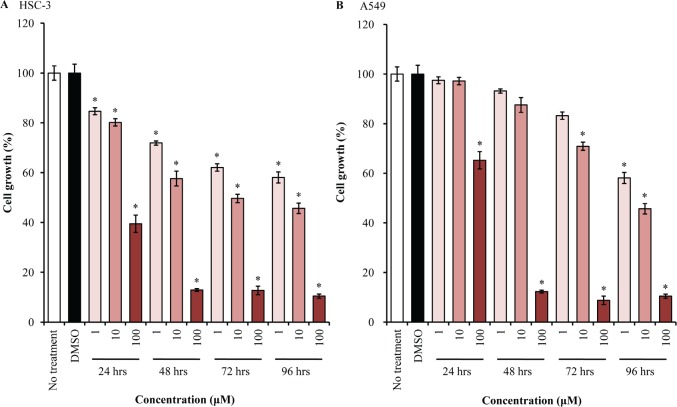
EGFR-TKI inhibits cell proliferation HSC-3 (A) and A549 (B) cells were treated with different concentrations (1-100 μM) of AG1478 for different durations (24-96 hrs). Then, cell proliferation was measured (*n* = 6), using a kit and absorbance at 530 nm or 630 nm. Bars represent average ± standard deviation (SD) of three independent experiments. *P < 0.001 by student's *t* test (*vs* DMSO-treated cells).

We first investigated the growth inhibition effect of EGFR-TKI treatment in human tongue and lung cancer cells, using MTT assay. AG1478 treatment could decrease the growth of HSC-3 cells dramatically in dose- and time-dependent manners, as compared with mock-treated cells (Figure 1A). The growth of A549 cells was similarly inhibited by AG1478 (Figure 1B).

**Figure 2:**
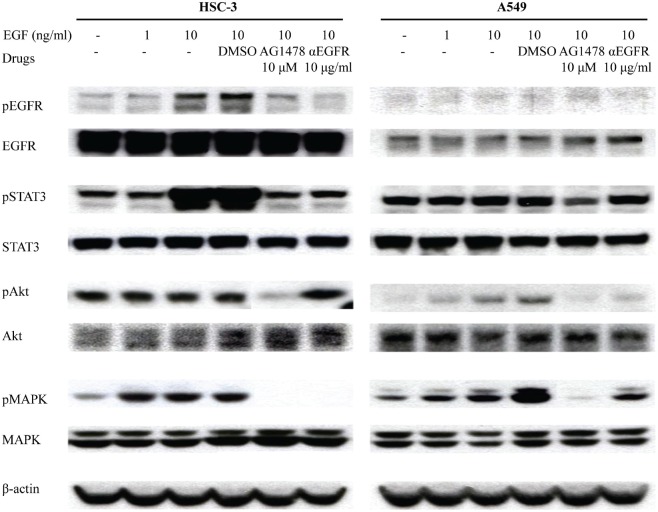
EGFR-TKI inhibits phosphorylation of molecules related to downstream signaling of EGFR

To confirm the inhibition of EGF pathway by EGFR-TKI treatment, HSC-3 and A549 cells were treated with EGF after the pre-treatment of AG1478 and αEGFR antibody. EGF treatment stimulated EGFR phosphorylation at 10 min (Figure 2). EGF treatment also increased phosphorylation of STAT3 and MAPK in HSC-3 cells as well as Akt phosphorylation in A549 cells. When cells were pre-treated with AG1478 or αEGFR antibody, EGFR phosphorylation was inhibited especially in HSC-3. AG1478 also inhibited phosphorylation of STAT3, Akt, and MAPK. These results suggest that EGFR-TKI and αEGFR antibody decrease cell growth via inhibiting EGF phosphorylation.

HSC-3 and A549 cells treated with EGF and other drugs as indicated were analyzed on western blotting, using antibodies to the downstream signaling of EGFR, including pEGFR (175 kDa), EGFR (175 kDa), pSTAT3 (86 kDa), STAT3 (86 kDa), pAKT (60 kDa), AKT (60 kDa), pMAPK (42 and 44 kDa), and MAPK (42 and 44 kDa). β-Actin (43 kDa) served as an internal control.

### Cancer cells treated with EGFR-TKI secretes IL-6

**Figure 3:**
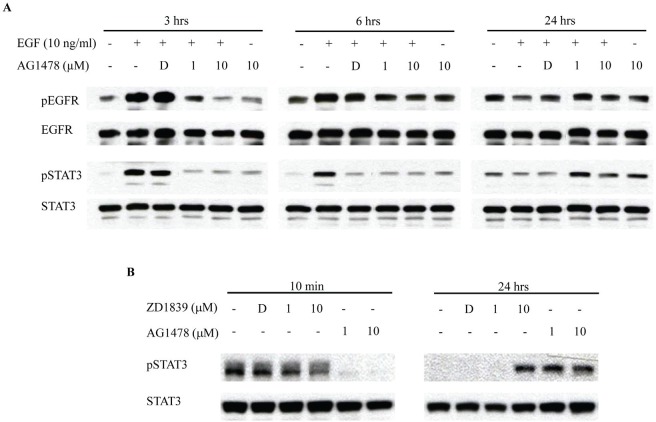
EGFR-TKI increases phosphorylation of STAT3

Western blotting was then performed in HSC-3 cells treated with AG1478 for up to 24 hrs. As shown in Figure 3A, EGF induced EGFR and STAT3 phosphorylation from 10 min to 6 hrs. Further, AG1478 pre-treatment effectively prevented their phosphorylation induced by EGF stimulation. On the other hand, AG1478 pre-treatment increased STAT3 phosphorylation at 24 hrs while EGF treatment did not induce the phosphorylation of EGFR and STAT3 at 24 hrs. We also confirmed STAT3 phosphorylation at 24 hrs using another EGFR-TKI, ZD1839 (Figure 3B).

(A) HSC-3 cells treated with EGF and/or AG1478 for up to 24 hrs were analyzed on western blotting, using antibodies to pEGFR (175 kDa), EGFR (175 kDa), pSTAT3 (86 kDa), and STAT3 (86 kDa). (B) HSC-3 cells treated with AG1478 or ZD1478 for 10 min or 24 hrs were analyzed on western blotting, using antibodies to pEGFR (175 kDa), EGFR (175 kDa), pSTAT3 (86 kDa), and STAT3 (86 kDa). D, DMSO.

**Figure 4:**
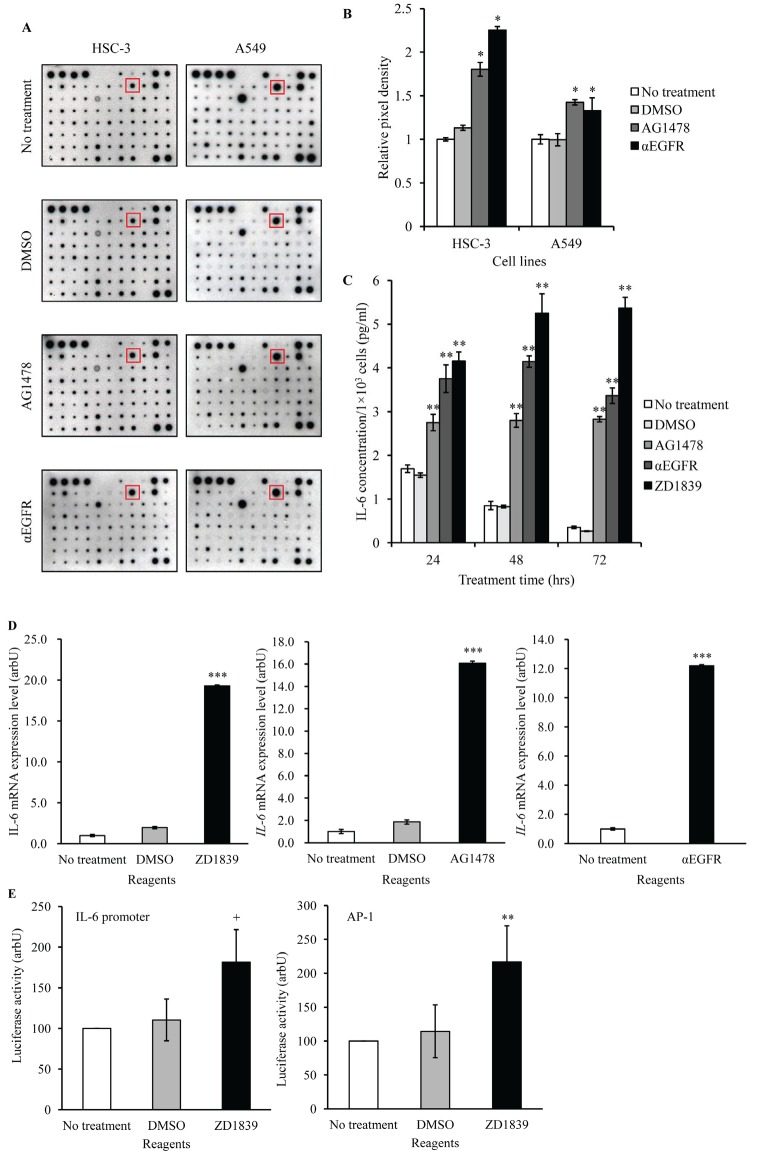
IL-6 is increased in cancer cells treated with EGFR-TKI

Growth factors or cytokines are well known to increase STAT3 phosphorylation [21]. Therefore, we anticipated that growth factors or cytokines could be induced by EGFR-TKI treatment. Using cytokine membrane arrays, we screened factors that were up- or down-regulated in Conditioned Medium (CM) obtained from EGFR-TKI- or αEGFR antibody-treated cancer cells. Of the factors, IL-6 was found to be augmented significantly in CM with EGFR-TKI and αEGFR antibody, compared with control CM. (Figure 4A and B). In addition, we examined IL-6 protein secretion by ELISA and mRNA expression by qPCR in HSC-3 cells. All of EGFR blocking reagents increased IL-6 secretion (Figure 4C). Similarly, these EGFR blocking reagents also increased *IL-6* mRNA in HSC-3 cells, compared with that in control- or DMSO-treated cells (Figure 4D). These results suggested increases in *IL-6* mRNA levels were regulated at a transcriptional level. Then, we tested the effects of EGFR blocking on IL-6 promoter transcriptional activity in HSC-3 cells, using luciferase reporter gene assay. Because the IL-6 promoter contains AP-1 binding site, AP-1 transcription was also assessed. As expected, ZD1839 treatment induced transcriptional activity of IL-6 and AP-1 (Figure 4E). Thus, EGFR blocking reagents appeared to activate IL-6 promoter transcription presumably via the regulation of AP-1 activation.

(A) HSC-3 and A549 cells were treated with AG1478 (10 μM) or αEGFR antibody(10 μg/ml) for 24 hrs. Then, cytokines and growth factors included in CM were assayed using cytokine membrane array. DMSO was used as a vehicle. (B) Detected signal density obtained from the results of cytokine membrane array was measured using ScionImage software. Positive control spots were used as the internal control. *P < 0.01 by student's *t* test (*vs* DMSO). (C) HSC-3 cells were treated with AG1478 (10 μM), αEGFR antibody (5 μg/ml), ZD1839 (10 μM) or DMSO for 24 hrs, and CM was collected and assayed using an IL-6 ELISA kit. **P < 0.001 by student's *t* test (*vs* DMSO). (D) HSC-3 cells were treated with AG1478 (10 μM), ZD1839 (10 μM), or αEGFR antibody (5 μg/ml). Then, IL-6 mRNA expression was measured using qPCR. GAPDH was used as the internal control. Bars represent average ± SD of three independent experiments. ***P < 0.001 by student's *t* test (*vs* DMSO or no treatment). arbU; arbitrary units. (E) HSC-3 cells transfected with pGL4-IL-6 promoter luciferase vector or AP-1 luciferase vector were treated with DMSO or ZD1839 (10 μM) for 48 hrs. Then, luciferase activity was assayed using the dual-luciferase reporter assay system. +P =0.01 and **P < 0.01 by student's *t* test (*vs* DMSO). arbU; arbitrary units.

### IL-6 increases COL1A1 expression in lung fibroblasts

**Figure 5:**
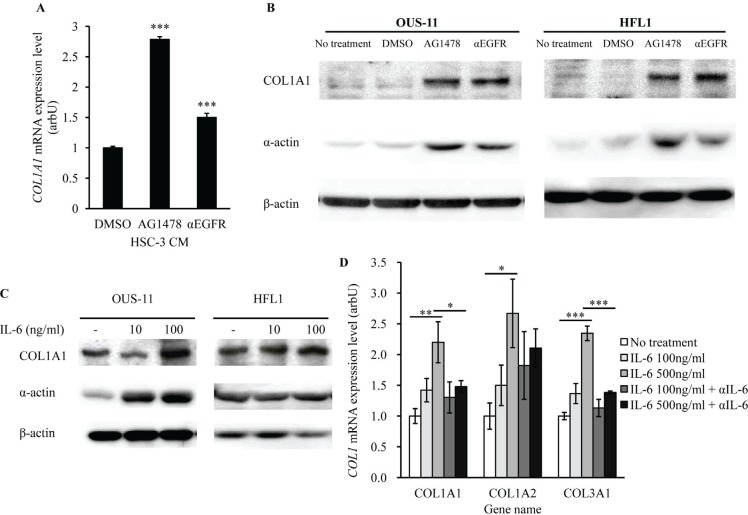
EGFR-TKI and IL-6 increase the expression of fibrosis markers

Because IL-6 has been linked to the development of acute interstitial pneumonia [15-18], we expected that EGFR-TKI treatment-induced IL-6 production in cancer cells resulted in lung fibrosis. To confirm this hypothesis, we assessed the expression of fibrosis markers in normal lung cells, COL1A1 and α-actin, in lung fibroblastic cells. CM obtained from HSC-3 cells treated with EGFR-TKI or αEGFR antibody was added to OUS-11 cell culture. The levels of *COL1A1* mRNA in OUS-11 cells were higher when treated with AG1478 or αEGFR antibody (Figure 5A). We also assessed COL1A1 and α-actin protein expression using the co-culture models (HSC-3/OUS-11, HSC-3/HEF1) followed by western blot. In both cell lines AG1478 or αEGFR antibody increased the levels of COL1A1 and α-actin proteins (Figure 5B). To confirm that IL-6 itself up-regulates COL1A1 and α-actin, we examined whether recombinant IL-6 increased COL1A1 expression. When OUS-11 and HFL1 cells were treated with IL-6, the expression of COL1A1 protein (Figure 5C) and *COL1A1* mRNA (Figure 5D) was significantly enhanced. In addition, αIL-6 antibody pre-treatment antagonized *COL1A1* mRNA expression in IL-6-treated OUS-11 cells (Figure 5D). Other Collagen 1A subtypes (*COL1A2* and *COL1A3*) were also up-regulated by IL-6 treatment, which was inhibited by αIL-6 antibody. These results suggest that IL-6 may play a key role in the development of EGFR-TKI-induced lung fibrosis.

(A) OUS-3 cells were cultured with CM obtained from AG1478- or αEGFR antibody -treated HSC-3 cells for 72 hrs. Then, COL1A1 mRNA expression was measured using qPCR. GAPDH was used as the internal control. Bars represent average ± SD of three independent experiments. ***P < 0.001 by student's *t* test (*vs* DMSO). arbU; arbitrary units. (B) OUS-11 and HFL1 cells were co-cultured with AG1478- or αEGFR antibody -treated HSC-3 cells for 7 days. Then, COL1A1 (210 kDa) and α-actin (42 kDa) proteins were assayed using western blotting. β-Actin was used as internal control. (C) OUS-11 and HFL1 cells treated with IL-6 were analyzed on western blotting, using antibodies to COL1A1 and α-actin. β-Actin was used as internal control. (D) OUS-11 cells treated with IL-6 and/or αIL-6 antibody for 72 hrs were assayed for qPCR to determine mRNA levels of COL1A1, COL1A2, and COL1A3. GAPDH was used as the internal control. Bars represent average ± SD of three independent experiments. *P < 0.01, **P < 0.01, and ***P < 0.001 by student's *t* test. arbU; arbitrary units.

## DISCUSSION

It has been shown that EGF promotes cancer cell proliferation, migration, and invasion. Therefore, EGFR is one of the molecular targets for the treatment of malignancies, such as non-small cell lung cancer. In this study, we confirmed that EGFR-TKI had anti-tumor effects in not only lung adenocarcinoma but also tongue squamous cell carcinoma. Interestingly, growth suppression was more significant in HSC-3 tongue cancer cells than in A549 lung cancer cells. Thus, EGFR-TKI might also be effective in patients with tongue cancer.

EGFR-TKI has been clinically used in cancer patients although it is occasionally associated with severe side effects, including acute interstitial pneumonia. Therefore, to use EGFR-TKI more safely, it is necessary to clarify the mechanism of acute interstitial pneumonia development induced by EGFR-TKI. We found that EGFR-TKI treatment in cancer cells increased IL-6 secretion, even if EGFR phosphorylation was completely blocked by EGFR-TKI. Furthermore, EGFR blocking, using an αEGFR antibody that neutralizes EGFR by competition with EGF on the external domain of EGFR, also resulted in increases in IL-6 production from cancer cells. Therefore, IL-6 production by EGFR blocking might be rather the general reaction by EGFR blocking than EGFR-TKI-specific reaction.

Consistent with previous findings [13], we showed the enhancement of *IL-6* mRNA in EFGR-TKI-treated cancer cells. Thus, IL-6 expression might be controlled at a transcriptional level. Moreover, we showed EGFR-TKI treatment activated IL-6 promoter in HSC-3 cells. For *IL-6* mRNA transcription, some transcriptional factors, such as NFκB or AP-1, are needed to be activated [22]. Takada *et* *al.* showed Fra-1 was involved in lung interstitial disease associated with EGFR-TKI treatment in lung cells [23]. Indeed, AP-1 was activated by EGFR-TKI in our reporter assays. Thus, AP-1 activation might be one of the mechanisms for IL-6 overexpression in cancer cells. Other possibilities include activated STAT3 induced IL-6 transcription through IL-6-STAT3 axis as a positive feedback loop [24]. Yao *et* *al.* indicated that TGFβ could increase IL-6 at a transcriptional level independent of EGFR activation [25], which may explain a mechanism for up-regulation of IL-6 promoter transcriptional activity by EGFR-TKI. Further investigations are needed to clarify how EGFR-TKI regulates IL-6 production in cancer cells.

**Figure 6:**
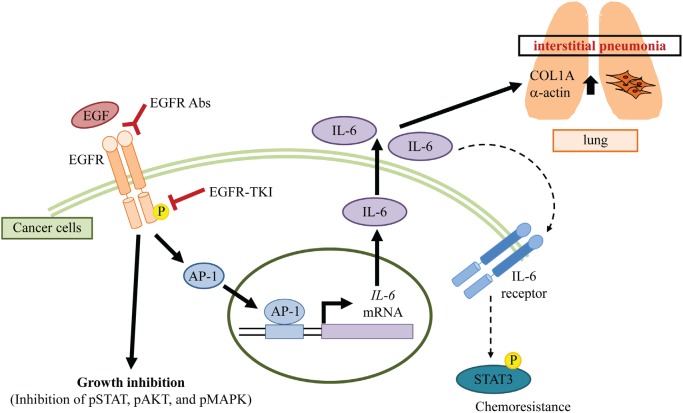
A potential mechanism of interstitial pneumonia induced by EGFR blocking

From our findings, IL-6 from cancer cells could induce COL1A1 and α-actin expression in lung fibroblast cells, suggesting IL-6 was likely one of the important factors for lung fibrosis. Because IL-6 strongly induces the growth and invasion of cancer cells [14,15], previous studies were focused on the relationship between IL-6 and anti-cancer treatment. On the other hand, IL-6 is also known to induce interstitial pneumonia [17-20]. Therefore, we focused on studying the role of IL-6 in the development of acute interstitial pneumonia induced by EGFR-TKI treatment. Other molecules, such as HSP70 [26] and TGFβ [27], have also been considered to involve interstitial pneumonia. Interestingly, these molecules are related to IL-6 expression [25,28,29]. Our results showed that αIL-6 antibody blocked COL1A1 expression. Thus, IL-6 from cancer cells may play a central role in EGFR-TKI-induced interstitial pneumonia. Taken together, we propose the following mechanism for the development of interstitial pneumonia as an adverse effect of EGFR-TKI treatment (Figure 6). EGFR-TKI reduces EGFR phosphorylation, which results in decreases in phosphorylation of some other molecules under the EGFR pathway. Consequently, the growth of cancer cells is suppressed. On the other hand, EGFR-TKI activates AP-1 in cancer cells and produces a large amount of IL-6 while growth inhibition is observed. Increased IL-6 affects both cancer and lung fibroblast cells. In cancer cells, IL-6 induces chemoresistance through IL-6-STAT3 axis. In non-neoplastic cells, IL-6 induces fibrosis. Thus, IL-6 production from EGFR-TKI-treated cancer cells increases the risk of chemoresistance and acute interstitial pneumonia.

We demonstrate *in* *vitro* evidence suggesting that IL-6 from cancer cells induces acute interstitial pneumonia as one of mechanisms responsible for the occurrence of a side effect of EGFR-TKI treatment. Furthermore, our findings suggest that the inhibition of IL-6-STAT3 axis in patients treated with EGFR-TKI could be not only a therapeutic target for their cancers but also an option to prevent the development of acute interstitial pneumonia as an adverse effect. Thus, EGFR-TKI along with an IL-6 inhibitor might be more effective and safer treatment in patients with malignancy.

## METHODS

### Cell lines

HSC-3 (human tongue squamous cell carcinoma), A549 (human lung adenocarcinoma), OUS-11 (normal human lung fibroblast-like cell), and HFL1 (human fetal lung fibroblast) cell lines were obtained from American Type Culture Collection (Manassas, VA, USA). ALL cell lines were authenticated in our lab and were used within 6 months after authentication. All the cell lines were cultured with appropriate medium (DMEM for HSC-3 and A549; MEM for OUS-11; and F-12K for HFL1) supplemented with 10% fetal bovine serum (FBS) under 5% CO_2_ at 37°C.

### Reagents and Antibodies

Recombinant human EGF, ZD1839 (EGFR-TKI), and anti-β-actin antibody were obtained from Sigma (St. Louis, MO, USA). AG1478 used as another EGFR-TKI was obtained from Calbiochem (San Diego, CA, USA). For neutralizing EGFR, αEGFR antibody (clone LA1) was obtained from Upstate Biotechnology (Lake Placid, NY, USA). Recombinant human IL-6 and neutralizing αIL-6 antibody were obtained from R&D Systems (Minneapolis, MN, USA). Anti-pEGFR, anti-pSTAT3, anti-STAT3, anti-pAkt, anti-Akt, anti-pMAPK, and anti-MAPK antibodies were purchased from Cell Signaling Technology (Danvers, MA, USA). Anti-EGFR, anti-collagen 1A1 (COL1A1), and anti-α-actin antibodies were purchased from Santa Cruz Biotechnology (Santa Cruz, CA, USA).

### Cell proliferation assay

Cells were seeded onto 96-well plate at a density of 1 × 10^3^ cells/well. Two days after seeding, the cells were treated with EGFR-TKI and incubated in 5% CO_2_ at 37C for 24-96 hrs. Cell growth was then measured, using TetraColor ONE (Seikagakukogyo, Tokyo, Japan), according to manufacturer's procedures. Absorbance was measured at 530 nm and 630 nm as references.

### Western blotting

HSC-3 and A549 cells were seeded onto 6-well plate at a density of 1 × 10^5^ cells/well. Two days after seeding, the cells were pretreated with EGFR-TKI or αEGFR antibody for 30 min and then treated with EGF. OUS-11 and HFL1 cells were seeded onto 6-well plate at a density of 1 × 10^5^ cells/well. At a day after seeding, the cells were pretreated with neutralizing αIL-6 antibody for 30 min and then treated with IL-6. These cells were washed twice with ice-cold phosphate-buffered saline (PBS) and lysed in ice-cold RIPA buffer. After boiling cell lysate (10 μg) with gel loading buffer for 5 min, the samples were subjected to SDS-PAGE on 7.5% gel and transferred to Immobilon-P (Millipore, Billerica, MA). After blocking the membrane with blocking reagent (NOF, Tokyo, Japan), western blotting was done using the antibody of interest, and the products were detected with Immobilon Western Chemiluminescent HRP substrate (Millipore).

### Cytokine Antibody array

Cytokines in CM were detected, using Human Cytokine Array V (Ray Biotech, Norcross, GA, USA), according to the manufacturer's instructions. HSC-3 or A549 (1 × 10^5^ cells seeded in 6-well plate and incubated for 48 hrs) were treated with EGFR-TKI or αEGFR antibody. After 24 hrs incubation, medium was collected as CM and CM was used for cytokine membrane assay. Detected signal density was measured by ScionImage Software.

### Quantification of IL-6 and COL1A1 mRNA by qPCR

HSC-3 or OUS-11 cells were seeded onto 6-well plate at a density of 1 × 10^5^ cells/well. One (for HSC-3) or two (for OUS-11) days after seeding, the cells were treated with EGFR-TKI or αEGFR antibody for 24 hrs, treated with CM for 72 hrs, or pretreated with αIL-6 antibody for 30 min and then treated with IL-6 for 72 hrs. After each treatment, total RNA was extracted using ISOGEN (Nippon Gene, Toyama, Japan) according to the manufacturer's instructions. cDNA was synthesized using High Capacity cDNA Reverse Transcription Kit (Life Technologies, Grand Island, NY). qPCR was assayed using TaqMan Fast Advanced Mastermix and TaqMan according to the manufacturer's protocol (Life Technologies). GAPDH was used as an internal control.

### Quantification of IL-6 secretion by ELISA

HSC-3 cells (1 × 10^5^ cells/well) were seeded onto 6-well plates. Two days after seeding, the cells were treated with EGFR-TKI or αEGFR antibody. After incubation, the culture supernatants were collected and the number of cells was counted. The levels of IL-6 were analyzed by human IL-6 ELISA kit II according to the manufacturer's protocol (BD Biosciences) and adjusted by the total number of cells.

### OUS-11 and HFL1 co-cultured with HSC-3

OUS-11 and HFL1 cells were seeded onto 6-well plate at a density of 1 × 10^5^ cells/well. Then, HSC-3 cells were seeded onto cell culture insert at a density of 1 × 10^4^ cells. Two days after seeding, cell culture insert was set on wells of the 6-well plates, and cultured with EGFR-TKI or αEGFR antibody for 7 days in 5% CO_2_ at 37°C. Thereafter, the cells were washed twice with ice-cold PBS and lysed in ice-cold RIPA buffer. Then, COL1A1 and α-actin expression was assayed by western blot.

### Luciferase assay

pGL4-IL-6 promoter luciferase vector was obtained as described in a previously report [30]. AP-1 luciferase vector was purchased from Clontech (Palo Alto, CA). phRL-SV40 (Promega, Madison, WI) was used as the internal control. HSC-3 cells were seeded onto 24-well plate at a density of 5 × 10^4^ cells. After the vectors were transfected using Transfast reagents (Promega), the cells were treated with drug and incubated for 48 hrs. Then, the cells were lysed and assayed using the dual-luciferase reporter assay system (Promega) and a luminometer, TD-20/20 (Turner Design, Sunnyvale, CA).
